# Aqua­{2,2-[ethane-1,2-diylbis(nitrilo­methyl­idyne)]diphenolato}(3-nitro­benzoato)manganese(III)

**DOI:** 10.1107/S1600536808019715

**Published:** 2008-07-05

**Authors:** V. S Thampidas, T. Radhakrishnan, Robert D. Pike

**Affiliations:** a Department of Chemistry, SN College, Varkala, Kerala 695 145, India; b Department of Chemistry, University of Kerala, Thiruvananthapuram, Kerala 695 581, India; c Department of Chemistry, College of William and Mary, PO Box 8795, Williamsburg, VA 23187-8795, USA

## Abstract

The title compound, [Mn(C_16_H_14_N_2_O_2_)(C_7_H_4_NO_4_)(H_2_O)], is a Jahn–Teller-distorted manganese(III) monomer with an octa­hedral geometry. The tetra­dentate Schiff base accommodates the Mn^III^ ion at the centre of a nearly planar square. The axial positions are occupied by a monodentate carboxyl­ate group and a water mol­ecule. Adjacent monomers inter­act through hydrogen bonding between the noncoordinated C=O group of the carboxyl­ate and the coordinated water mol­ecule to produce chains extending parallel to the *b* axis.

## Related literature

For related literature, see: Christou (1989 ▶); Pecoraro & Hsieh (2000 ▶); Yocum & Pecoraro (2004 ▶); Zhang & Janiak (2001 ▶); Zouni *et al.* (2001 ▶); Aurangzeb *et al.* (1994 ▶); Hulme *et al.* (1997 ▶).
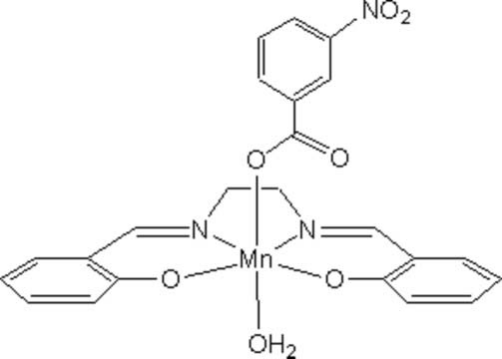


## Experimental

### 

#### Crystal data

[Mn(C_16_H_14_N_2_O_2_)(C_7_H_4_NO_4_)(H_2_O)]
*M* *_r_* = 505.36
Monoclinic, 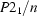

*a* = 6.7297 (1) Å
*b* = 10.5793 (2) Å
*c* = 29.228 (5) Å
β = 95.188 (1)°
*V* = 2072.4 (4) Å^3^
*Z* = 4
Cu *K*α radiation
μ = 5.66 mm^−1^
*T* = 100 (2) K
0.35 × 0.29 × 0.09 mm

#### Data collection

Bruker SMART APEXII CCD diffractometer
Absorption correction: multi-scan (*SADABS*; Sheldrick, 2004 ▶) *T*_min_ = 0.245, *T*_max_ = 0.624
21257 measured reflections
3640 independent reflections
3492 reflections with *I* > 2σ(*I*)
*R*_int_ = 0.046

#### Refinement

*R*[*F* ^2^ > 2σ(*F* ^2^)] = 0.035
*wR*(*F* ^2^) = 0.092
*S* = 1.11
3640 reflections
308 parameters
H-atom parameters constrained
Δρ_max_ = 0.35 e Å^−3^
Δρ_min_ = −0.51 e Å^−3^

### 

Data collection: *APEX2* (Bruker, 2004 ▶); cell refinement: *SAINT-Plus* (Bruker, 2004 ▶); data reduction: *SAINT-Plus*; program(s) used to solve structure: *SHELXS97* (Sheldrick, 2008 ▶); program(s) used to refine structure: *SHELXL97* (Sheldrick, 2008 ▶) and *XSHELL* (Bruker, 2004 ▶); molecular graphics: *ORTEP-3* (Farrugia, 1997 ▶) and *Mercury* (Macrae *et al.* 2006 ▶); software used to prepare material for publication: *SHELXL97*.

## Supplementary Material

Crystal structure: contains datablocks global, I. DOI: 10.1107/S1600536808019715/si2096sup1.cif

Structure factors: contains datablocks I. DOI: 10.1107/S1600536808019715/si2096Isup2.hkl

Additional supplementary materials:  crystallographic information; 3D view; checkCIF report

## Figures and Tables

**Table:** 

Mn1—O1	1.8879 (15)
Mn1—O2	1.9113 (15)
Mn1—N2	1.9946 (18)
Mn1—N1	1.9980 (18)
Mn1—O3	2.1513 (15)
Mn1—O7	2.3250 (16)

**Table:** 

O1—Mn1—O2	97.25 (7)
O1—Mn1—N2	171.26 (7)
O2—Mn1—N2	91.15 (7)
O1—Mn1—N1	90.20 (7)
O2—Mn1—N1	172.54 (7)
N2—Mn1—N1	81.42 (7)
O1—Mn1—O3	91.05 (6)
O2—Mn1—O3	89.70 (6)
N2—Mn1—O3	91.40 (7)
N1—Mn1—O3	89.75 (7)
O1—Mn1—O7	90.07 (6)
O2—Mn1—O7	91.54 (6)
N2—Mn1—O7	87.29 (6)
N1—Mn1—O7	88.85 (6)
O3—Mn1—O7	178.21 (6)

**Table 2:** Hydrogen-bond geometry (Å, °)

*D*—H⋯*A*	*D*—H	H⋯*A*	*D*⋯*A*	*D*—H⋯*A*
O7—H2*W*⋯O2^i^	0.77	2.31	3.074 (2)	172
O7—H1*W*⋯O4^ii^	0.84	1.89	2.710 (2)	166

Symmetry codes: (i) 
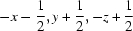
; (ii) 
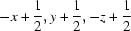
.

**Fig. 1.**
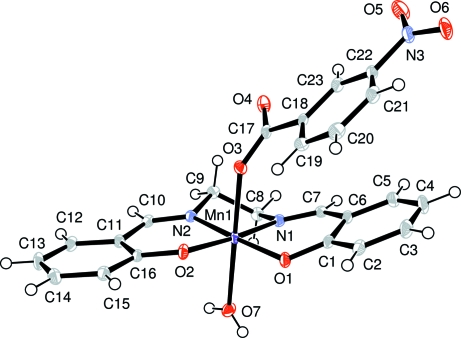
ORTEP picture (Farrugia, 1997) of the title compound. Displacement ellipsoids have been drawn at the 50% probability level.

**Fig. 2.**
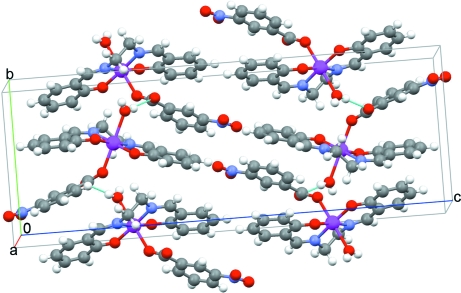
Mercury (Macrae et al. 2006) ball and stick packing diagram of the title compound showing hydrogen-bonding chains.

## supplementary crystallographic information

### Comment

Manganese plays a vital role in several biological systems like the oxygen-evolving complex (OEC) of photosystem II (Zouni *et al.*, 2001) and enzymes like superoxide dismutase, catalase, arginase etc. (Yocum & Pecoraro, 2004). The progress in elucidating the structural and functional aspects of the active-sites of these manganese-containing systems, has essentially been connected to the vast number of inorganic model complexes reported during the last few decades (Christou, 1989; Pecoraro & Hsieh, 2000). Recent reports include a few Schiff base complexes of manganese(III) with ancillary carboxylate ligands (Aurangzeb *et al.*, 1994, Hulme *et al.*, 1997; Zhang & Janiak, 2001). Generally, symmetrical N_2_O_2_ Schiff base ligands like the salen(H_2_salen = N,N^'^-bis(salicylidene)-1,2-diaminoethane), with aromatic rings amenable to π-π overlap, tend to stabilize µ-phenoxy dimers. Here we report a very rare structural type among such complexes, where the trans coordination positions in an octahedral manganese(III) monomer are occupied by a neutral ligand (H_2_O) and a monodentate carboxylate group (Fig. 1).

In the title compound, the salen ligand holds the manganese(III) ion at the centre of a nearly planar tetragon consisting of two Mn–O bonds [Mn(1)-O(1) = 1.8879 (15) Å and Mn(1)-O(2) = 1.9113 (15) Å] and two Mn–N bonds [Mn(1)-N(1) = 1.9980 (18) Å and Mn(1)-N(2) = 1.9946 (18)Å]. These bond lengths are comparable to those in complexes containing similar MnN_2_O_2_ cores (Aurangzeb *et al.*, 1994; Hulme *et al.*, 1997). Jahn-Teller distortion causes an elongation of the Mn–Ocarb [Mn(1)-O(3) = 2.1513 (15) Å] and the Mn–Oaq [Mn(1)-O(7) = 2.3250 (16) Å] axial bonds (Table 1). Chains running parallel to the *b*-axis arise from H-bonding interactions between the non-coordinated O atom of the carboxylate and coordinated water molecules on adjacent molecules (Fig.2, Table 2).

### Experimental

To a solution of Mn(m—NO_2_C_6_H_4_CO_2_)_2_.2H_2_O (1.00 g, 2.36 mmol) and salicylaldehyde (0.58 g, 4.72 mmol) in methanol (40 ml), ethane-1,2-diamine (0.14 g, 2.36 mmol) was added. The solution was stirred for 20 minutes, filtered and left to evaporation in an open conical flask. Brown crystals were deposited in 2–3 days. These were collected by filtration, washed with methanol, and dried in air. Yield of the title compound was 0.82 g (75.50%) based on manganese.

### Refinement

All hydrogen atoms were initially located in the difference map and then were placed in theoretical positions using a riding model for all but the water H atoms which were allowed to rotate freely, O–H = 0.77 and 0.84 Å. C*sp*^2^–H = 0.95 Å, C*sp*^3^–H = 0.99 Å, *U*_iso_(H) = 1.2*U*_eq_(C,O).

### Crystal data

**Table:**

[Mn(C_16_H_14_N_2_O_2_)(C_7_H_4_NO_4_)(H_2_O)]	*F*_000_ = 1040
*M**_r_* = 505.36	*D*_x_ = 1.620 Mg m^−^^3^
Monoclinic, *P*2_1_/*n*	Cu *K*α radiation λ = 1.54178 Å
*a* = 6.7297 (1) Å	Cell parameters from 256 reflections
*b* = 10.5793 (2) Å	θ = 9.7–70.9º
*c* = 29.228 (5) Å	µ = 5.66 mm^−^^1^
β = 95.188 (1)º	*T* = 100 (2) K
*V* = 2072.4 (4) Å^3^	Plate, brown
*Z* = 4	0.35 × 0.29 × 0.09 mm

### Data collection

**Table:**

Bruker SMART APEXII CCD diffractometer	3640 independent reflections
Radiation source: fine-focus sealed tube	3492 reflections with *I* > 2σ(*I*)
Monochromator: graphite	*R*_int_ = 0.046
*T* = 100(2) K	θ_max_ = 67.0º
ω and ψ scans	θ_min_ = 3.0º
Absorption correction: multi-scan(SADABS; Sheldrick, 2004)	*h* = −7→7
*T*_min_ = 0.245, *T*_max_ = 0.624	*k* = −10→12
21257 measured reflections	*l* = −31→34

### Refinement

**Table:**

Refinement on *F*^2^	Secondary atom site location: difference Fourier map
Least-squares matrix: full	Hydrogen site location: inferred from neighbouring sites
*R*[*F*^2^ > 2σ(*F*^2^)] = 0.035	H-atom parameters constrained
*wR*(*F*^2^) = 0.092	*w* = 1/[σ^2^(*F*_o_^2^) + (0.0418*P*)^2^ + 2.1055*P*] where *P* = (*F*_o_^2^ + 2*F*_c_^2^)/3
*S* = 1.11	(Δ/σ)_max_ < 0.001
3640 reflections	Δρ_max_ = 0.35 e Å^−^^3^
308 parameters	Δρ_min_ = −0.51 e Å^−^^3^
Primary atom site location: structure-invariant direct methods	Extinction correction: none

### Special details

**Table:**

Geometry. All e.s.d.'s (except the e.s.d. in the dihedral angle between two l.s. planes) are estimated using the full covariance matrix. The cell e.s.d.'s are taken into account individually in the estimation of e.s.d.'s in distances, angles and torsion angles; correlations between e.s.d.'s in cell parameters are only used when they are defined by crystal symmetry. An approximate (isotropic) treatment of cell e.s.d.'s is used for estimating e.s.d.'s involving l.s. planes.
Refinement. Refinement of *F*^2^ against ALL reflections. The weighted *R*-factor *wR* and goodness of fit *S* are based on *F*^2^, conventional *R*-factors *R* are based on *F*, with *F* set to zero for negative *F*^2^. The threshold expression of *F*^2^ > σ(*F*^2^) is used only for calculating *R*-factors(gt) *etc*. and is not relevant to the choice of reflections for refinement. *R*-factors based on *F*^2^ are statistically about twice as large as those based on *F*, and *R*- factors based on ALL data will be even larger.

### Fractional atomic coordinates and isotropic or equivalent isotropic displacement parameters (Å^2^)

**Table:**

	*x*	*y*	*z*	*U*_iso_*/*U*_eq_	
Mn1	−0.00146 (5)	0.54194 (3)	0.229187 (11)	0.01142 (12)	
O1	−0.2188 (2)	0.55831 (14)	0.18400 (5)	0.0155 (3)	
O2	−0.1396 (2)	0.45279 (14)	0.27355 (5)	0.0147 (3)	
O3	0.0816 (2)	0.36457 (14)	0.20036 (5)	0.0171 (3)	
O4	0.3542 (2)	0.32126 (15)	0.16477 (5)	0.0197 (4)	
O5	0.2710 (3)	0.15274 (18)	0.00870 (6)	0.0311 (4)	
O6	−0.0245 (3)	0.12490 (17)	−0.02568 (5)	0.0282 (4)	
O7	−0.0810 (2)	0.73579 (15)	0.26027 (5)	0.0190 (3)	
H1W	0.0052	0.7545	0.2818	0.023*	
H2W	−0.1592	0.7863	0.2531	0.023*	
N1	0.1747 (3)	0.63243 (16)	0.18849 (6)	0.0133 (4)	
N2	0.2479 (3)	0.54511 (16)	0.27161 (6)	0.0129 (4)	
N3	0.0896 (3)	0.15170 (18)	0.00848 (6)	0.0220 (5)	
C1	−0.2066 (3)	0.5574 (2)	0.13906 (8)	0.0149 (5)	
C2	−0.3747 (3)	0.5194 (2)	0.11019 (8)	0.0171 (5)	
H2	−0.4930	0.4950	0.1233	0.020*	
C3	−0.3700 (4)	0.5173 (2)	0.06299 (8)	0.0223 (5)	
H3	−0.4848	0.4907	0.0442	0.027*	
C4	−0.1991 (4)	0.5536 (2)	0.04252 (8)	0.0239 (5)	
H4	−0.1976	0.5519	0.0101	0.029*	
C5	−0.0333 (4)	0.5917 (2)	0.06989 (8)	0.0202 (5)	
H5	0.0831	0.6167	0.0561	0.024*	
C6	−0.0331 (3)	0.5945 (2)	0.11816 (7)	0.0150 (4)	
C7	0.1445 (3)	0.63864 (19)	0.14441 (7)	0.0141 (4)	
H7	0.2464	0.6748	0.1281	0.017*	
C8	0.3601 (3)	0.6801 (2)	0.21297 (7)	0.0159 (5)	
H8A	0.3362	0.7622	0.2278	0.019*	
H8B	0.4639	0.6920	0.1914	0.019*	
C9	0.4256 (3)	0.5815 (2)	0.24879 (7)	0.0153 (4)	
H9A	0.4818	0.5070	0.2341	0.018*	
H9B	0.5291	0.6168	0.2715	0.018*	
C10	0.2631 (3)	0.5163 (2)	0.31447 (7)	0.0138 (4)	
H10	0.3899	0.5276	0.3310	0.017*	
C11	0.1053 (3)	0.46869 (19)	0.33948 (7)	0.0143 (4)	
C12	0.1515 (4)	0.4424 (2)	0.38667 (8)	0.0173 (5)	
H12	0.2813	0.4611	0.4006	0.021*	
C13	0.0131 (4)	0.3906 (2)	0.41291 (7)	0.0197 (5)	
H13	0.0454	0.3749	0.4447	0.024*	
C14	−0.1764 (4)	0.3614 (2)	0.39192 (8)	0.0184 (5)	
H14	−0.2734	0.3258	0.4098	0.022*	
C15	−0.2250 (3)	0.3835 (2)	0.34569 (7)	0.0157 (4)	
H15	−0.3541	0.3615	0.3322	0.019*	
C16	−0.0877 (3)	0.43777 (19)	0.31824 (7)	0.0134 (4)	
C17	0.1704 (3)	0.32348 (19)	0.16688 (7)	0.0142 (4)	
C18	0.0387 (3)	0.26862 (19)	0.12698 (7)	0.0140 (4)	
C19	−0.1613 (3)	0.2428 (2)	0.13095 (8)	0.0173 (5)	
H19	−0.2185	0.2639	0.1585	0.021*	
C20	−0.2791 (4)	0.1861 (2)	0.09498 (8)	0.0200 (5)	
H20	−0.4151	0.1676	0.0984	0.024*	
C21	−0.1988 (4)	0.1569 (2)	0.05438 (8)	0.0197 (5)	
H21	−0.2780	0.1188	0.0296	0.024*	
C22	−0.0006 (3)	0.1846 (2)	0.05094 (7)	0.0166 (5)	
C23	0.1219 (3)	0.2390 (2)	0.08636 (7)	0.0155 (4)	
H23	0.2586	0.2556	0.0830	0.019*	

### Atomic displacement parameters (Å^2^)

**Table:**

	*U*^11^	*U*^22^	*U*^33^	*U*^12^	*U*^13^	*U*^23^
Mn1	0.0138 (2)	0.01245 (19)	0.00759 (19)	−0.00076 (13)	−0.00106 (13)	0.00132 (12)
O1	0.0173 (8)	0.0190 (8)	0.0097 (8)	0.0005 (6)	−0.0012 (6)	0.0015 (6)
O2	0.0182 (8)	0.0155 (8)	0.0099 (7)	−0.0004 (6)	−0.0023 (6)	0.0019 (6)
O3	0.0262 (9)	0.0129 (8)	0.0121 (8)	0.0011 (6)	0.0018 (6)	−0.0013 (6)
O4	0.0206 (9)	0.0241 (9)	0.0137 (8)	−0.0041 (7)	−0.0018 (6)	0.0007 (6)
O5	0.0317 (11)	0.0424 (11)	0.0198 (9)	0.0022 (8)	0.0059 (7)	−0.0056 (8)
O6	0.0443 (11)	0.0275 (9)	0.0113 (8)	−0.0043 (8)	−0.0053 (7)	−0.0036 (7)
O7	0.0211 (9)	0.0181 (8)	0.0161 (8)	0.0047 (6)	−0.0063 (6)	−0.0028 (6)
N1	0.0163 (9)	0.0107 (9)	0.0128 (9)	0.0004 (7)	0.0004 (7)	0.0000 (7)
N2	0.0162 (10)	0.0096 (9)	0.0128 (9)	0.0004 (7)	−0.0003 (7)	−0.0009 (7)
N3	0.0357 (13)	0.0190 (10)	0.0109 (10)	0.0011 (9)	−0.0007 (8)	−0.0005 (8)
C1	0.0208 (12)	0.0104 (10)	0.0130 (11)	0.0025 (8)	−0.0016 (9)	0.0014 (8)
C2	0.0195 (12)	0.0153 (11)	0.0156 (11)	0.0006 (9)	−0.0025 (9)	0.0019 (9)
C3	0.0275 (13)	0.0222 (12)	0.0153 (12)	0.0000 (10)	−0.0081 (9)	−0.0016 (9)
C4	0.0343 (14)	0.0269 (13)	0.0096 (11)	0.0018 (10)	−0.0026 (10)	−0.0007 (9)
C5	0.0283 (13)	0.0180 (12)	0.0146 (11)	0.0027 (10)	0.0036 (9)	0.0015 (9)
C6	0.0224 (12)	0.0106 (10)	0.0117 (10)	0.0024 (9)	−0.0010 (8)	0.0009 (8)
C7	0.0201 (12)	0.0076 (10)	0.0151 (11)	0.0013 (8)	0.0042 (8)	0.0016 (8)
C8	0.0192 (11)	0.0124 (11)	0.0160 (11)	−0.0031 (8)	0.0008 (9)	−0.0005 (8)
C9	0.0142 (11)	0.0150 (11)	0.0165 (11)	−0.0004 (9)	0.0001 (8)	−0.0003 (9)
C10	0.0172 (11)	0.0099 (10)	0.0131 (11)	0.0003 (8)	−0.0042 (8)	−0.0022 (8)
C11	0.0215 (12)	0.0105 (10)	0.0106 (11)	0.0011 (8)	−0.0011 (8)	−0.0017 (8)
C12	0.0244 (12)	0.0136 (11)	0.0128 (11)	0.0005 (9)	−0.0043 (9)	−0.0009 (8)
C13	0.0337 (14)	0.0159 (11)	0.0088 (10)	−0.0018 (10)	−0.0022 (9)	0.0000 (8)
C14	0.0281 (13)	0.0134 (11)	0.0141 (11)	−0.0028 (9)	0.0046 (9)	−0.0009 (8)
C15	0.0201 (11)	0.0128 (10)	0.0140 (11)	0.0004 (9)	−0.0002 (8)	−0.0010 (8)
C16	0.0222 (12)	0.0083 (10)	0.0093 (10)	0.0026 (8)	−0.0001 (8)	−0.0011 (8)
C17	0.0230 (13)	0.0092 (10)	0.0100 (10)	−0.0020 (8)	−0.0008 (8)	0.0021 (8)
C18	0.0215 (12)	0.0091 (10)	0.0108 (10)	0.0015 (8)	−0.0024 (8)	0.0013 (8)
C19	0.0218 (12)	0.0135 (11)	0.0166 (11)	0.0022 (9)	0.0022 (9)	0.0004 (8)
C20	0.0165 (11)	0.0191 (12)	0.0237 (12)	−0.0005 (9)	−0.0024 (9)	0.0009 (9)
C21	0.0260 (13)	0.0158 (11)	0.0158 (11)	−0.0019 (9)	−0.0073 (9)	−0.0013 (9)
C22	0.0260 (12)	0.0124 (11)	0.0108 (10)	0.0019 (9)	−0.0016 (9)	−0.0003 (8)
C23	0.0191 (11)	0.0129 (10)	0.0140 (11)	−0.0006 (8)	−0.0018 (8)	0.0014 (8)

### Geometric parameters (Å, °)

**Table:**

Mn1—O1	1.8879 (15)	C7—H7	0.9500
Mn1—O2	1.9113 (15)	C8—C9	1.515 (3)
Mn1—N2	1.9946 (18)	C8—H8A	0.9900
Mn1—N1	1.9980 (18)	C8—H8B	0.9900
Mn1—O3	2.1513 (15)	C9—H9A	0.9900
Mn1—O7	2.3250 (16)	C9—H9B	0.9900
O1—C1	1.324 (3)	C10—C11	1.434 (3)
O2—C16	1.331 (3)	C10—H10	0.9500
O3—C17	1.269 (3)	C11—C12	1.414 (3)
O4—C17	1.244 (3)	C11—C16	1.426 (3)
O5—N3	1.220 (3)	C12—C13	1.373 (3)
O6—N3	1.236 (3)	C12—H12	0.9500
O7—H1W	0.8400	C13—C14	1.399 (3)
O7—H2W	0.7659	C13—H13	0.9500
N1—C7	1.288 (3)	C14—C15	1.381 (3)
N1—C8	1.471 (3)	C14—H14	0.9500
N2—C10	1.284 (3)	C15—C16	1.400 (3)
N2—C9	1.472 (3)	C15—H15	0.9500
N3—C22	1.472 (3)	C17—C18	1.515 (3)
C1—C2	1.408 (3)	C18—C19	1.389 (3)
C1—C6	1.421 (3)	C18—C23	1.393 (3)
C2—C3	1.383 (3)	C19—C20	1.394 (3)
C2—H2	0.9500	C19—H19	0.9500
C3—C4	1.397 (4)	C20—C21	1.383 (3)
C3—H3	0.9500	C20—H20	0.9500
C4—C5	1.374 (3)	C21—C22	1.378 (3)
C4—H4	0.9500	C21—H21	0.9500
C5—C6	1.411 (3)	C22—C23	1.389 (3)
C5—H5	0.9500	C23—H23	0.9500
C6—C7	1.439 (3)		
			
O1—Mn1—O2	97.25 (7)	C9—C8—H8A	110.5
O1—Mn1—N2	171.26 (7)	N1—C8—H8B	110.5
O2—Mn1—N2	91.15 (7)	C9—C8—H8B	110.5
O1—Mn1—N1	90.20 (7)	H8A—C8—H8B	108.7
O2—Mn1—N1	172.54 (7)	N2—C9—C8	107.21 (17)
N2—Mn1—N1	81.42 (7)	N2—C9—H9A	110.3
O1—Mn1—O3	91.05 (6)	C8—C9—H9A	110.3
O2—Mn1—O3	89.70 (6)	N2—C9—H9B	110.3
N2—Mn1—O3	91.40 (7)	C8—C9—H9B	110.3
N1—Mn1—O3	89.75 (7)	H9A—C9—H9B	108.5
O1—Mn1—O7	90.07 (6)	N2—C10—C11	125.7 (2)
O2—Mn1—O7	91.54 (6)	N2—C10—H10	117.2
N2—Mn1—O7	87.29 (6)	C11—C10—H10	117.2
N1—Mn1—O7	88.85 (6)	C12—C11—C16	119.4 (2)
O3—Mn1—O7	178.21 (6)	C12—C11—C10	117.3 (2)
C1—O1—Mn1	125.44 (14)	C16—C11—C10	123.1 (2)
C16—O2—Mn1	128.63 (14)	C13—C12—C11	121.5 (2)
C17—O3—Mn1	139.29 (14)	C13—C12—H12	119.2
Mn1—O7—H1W	109.5	C11—C12—H12	119.2
Mn1—O7—H2W	133.3	C12—C13—C14	118.7 (2)
H1W—O7—H2W	116.8	C12—C13—H13	120.6
C7—N1—C8	121.31 (19)	C14—C13—H13	120.6
C7—N1—Mn1	124.86 (15)	C15—C14—C13	121.2 (2)
C8—N1—Mn1	113.44 (13)	C15—C14—H14	119.4
C10—N2—C9	120.50 (19)	C13—C14—H14	119.4
C10—N2—Mn1	126.20 (16)	C14—C15—C16	121.3 (2)
C9—N2—Mn1	113.27 (13)	C14—C15—H15	119.3
O5—N3—O6	123.7 (2)	C16—C15—H15	119.3
O5—N3—C22	118.83 (19)	O2—C16—C15	118.9 (2)
O6—N3—C22	117.5 (2)	O2—C16—C11	123.2 (2)
O1—C1—C2	118.6 (2)	C15—C16—C11	117.8 (2)
O1—C1—C6	123.5 (2)	O4—C17—O3	125.8 (2)
C2—C1—C6	117.9 (2)	O4—C17—C18	118.00 (19)
C3—C2—C1	120.9 (2)	O3—C17—C18	116.17 (19)
C3—C2—H2	119.6	C19—C18—C23	119.6 (2)
C1—C2—H2	119.6	C19—C18—C17	121.01 (19)
C2—C3—C4	121.1 (2)	C23—C18—C17	119.3 (2)
C2—C3—H3	119.4	C18—C19—C20	120.8 (2)
C4—C3—H3	119.4	C18—C19—H19	119.6
C5—C4—C3	119.2 (2)	C20—C19—H19	119.6
C5—C4—H4	120.4	C21—C20—C19	120.3 (2)
C3—C4—H4	120.4	C21—C20—H20	119.9
C4—C5—C6	121.0 (2)	C19—C20—H20	119.9
C4—C5—H5	119.5	C22—C21—C20	118.1 (2)
C6—C5—H5	119.5	C22—C21—H21	121.0
C5—C6—C1	119.9 (2)	C20—C21—H21	121.0
C5—C6—C7	117.7 (2)	C21—C22—C23	123.2 (2)
C1—C6—C7	122.4 (2)	C21—C22—N3	119.2 (2)
N1—C7—C6	124.4 (2)	C23—C22—N3	117.6 (2)
N1—C7—H7	117.8	C22—C23—C18	118.1 (2)
C6—C7—H7	117.8	C22—C23—H23	121.0
N1—C8—C9	106.32 (17)	C18—C23—H23	121.0
N1—C8—H8A	110.5		
			
O2—Mn1—O1—C1	−146.55 (16)	C2—C1—C6—C7	178.0 (2)
N2—Mn1—O1—C1	49.5 (5)	C8—N1—C7—C6	−179.76 (19)
N1—Mn1—O1—C1	33.04 (17)	Mn1—N1—C7—C6	7.9 (3)
O3—Mn1—O1—C1	−56.72 (16)	C5—C6—C7—N1	−171.5 (2)
O7—Mn1—O1—C1	121.89 (16)	C1—C6—C7—N1	10.2 (3)
O1—Mn1—O2—C16	−161.93 (17)	C7—N1—C8—C9	−136.1 (2)
N2—Mn1—O2—C16	15.66 (17)	Mn1—N1—C8—C9	37.1 (2)
N1—Mn1—O2—C16	21.3 (6)	C10—N2—C9—C8	−145.69 (19)
O3—Mn1—O2—C16	107.05 (17)	Mn1—N2—C9—C8	35.9 (2)
O7—Mn1—O2—C16	−71.66 (17)	N1—C8—C9—N2	−45.6 (2)
O1—Mn1—O3—C17	78.3 (2)	C9—N2—C10—C11	−174.05 (19)
O2—Mn1—O3—C17	175.5 (2)	Mn1—N2—C10—C11	4.1 (3)
N2—Mn1—O3—C17	−93.3 (2)	N2—C10—C11—C12	180.0 (2)
N1—Mn1—O3—C17	−11.9 (2)	N2—C10—C11—C16	5.2 (3)
O7—Mn1—O3—C17	−50.6 (19)	C16—C11—C12—C13	−1.6 (3)
O1—Mn1—N1—C7	−24.13 (18)	C10—C11—C12—C13	−176.7 (2)
O2—Mn1—N1—C7	152.7 (5)	C11—C12—C13—C14	1.2 (3)
N2—Mn1—N1—C7	158.36 (18)	C12—C13—C14—C15	0.2 (3)
O3—Mn1—N1—C7	66.92 (18)	C13—C14—C15—C16	−1.0 (3)
O7—Mn1—N1—C7	−114.20 (18)	Mn1—O2—C16—C15	171.22 (14)
O1—Mn1—N1—C8	162.98 (14)	Mn1—O2—C16—C11	−11.9 (3)
O2—Mn1—N1—C8	−20.2 (6)	C14—C15—C16—O2	177.58 (19)
N2—Mn1—N1—C8	−14.53 (14)	C14—C15—C16—C11	0.5 (3)
O3—Mn1—N1—C8	−105.98 (14)	C12—C11—C16—O2	−176.14 (19)
O7—Mn1—N1—C8	72.91 (14)	C10—C11—C16—O2	−1.4 (3)
O1—Mn1—N2—C10	152.3 (4)	C12—C11—C16—C15	0.8 (3)
O2—Mn1—N2—C10	−11.72 (18)	C10—C11—C16—C15	175.50 (19)
N1—Mn1—N2—C10	169.01 (19)	Mn1—O3—C17—O4	77.7 (3)
O3—Mn1—N2—C10	−101.45 (18)	Mn1—O3—C17—C18	−104.5 (2)
O7—Mn1—N2—C10	79.77 (18)	O4—C17—C18—C19	165.9 (2)
O1—Mn1—N2—C9	−29.4 (5)	O3—C17—C18—C19	−12.1 (3)
O2—Mn1—N2—C9	166.56 (14)	O4—C17—C18—C23	−11.5 (3)
N1—Mn1—N2—C9	−12.70 (14)	O3—C17—C18—C23	170.54 (19)
O3—Mn1—N2—C9	76.84 (14)	C23—C18—C19—C20	0.8 (3)
O7—Mn1—N2—C9	−101.95 (14)	C17—C18—C19—C20	−176.6 (2)
Mn1—O1—C1—C2	154.51 (16)	C18—C19—C20—C21	−1.2 (3)
Mn1—O1—C1—C6	−26.4 (3)	C19—C20—C21—C22	0.4 (3)
O1—C1—C2—C3	179.8 (2)	C20—C21—C22—C23	0.7 (3)
C6—C1—C2—C3	0.6 (3)	C20—C21—C22—N3	178.6 (2)
C1—C2—C3—C4	−0.5 (4)	O5—N3—C22—C21	−165.6 (2)
C2—C3—C4—C5	0.1 (4)	O6—N3—C22—C21	14.7 (3)
C3—C4—C5—C6	0.1 (4)	O5—N3—C22—C23	12.4 (3)
C4—C5—C6—C1	0.0 (3)	O6—N3—C22—C23	−167.3 (2)
C4—C5—C6—C7	−178.4 (2)	C21—C22—C23—C18	−1.0 (3)
O1—C1—C6—C5	−179.4 (2)	N3—C22—C23—C18	−178.95 (18)
C2—C1—C6—C5	−0.3 (3)	C19—C18—C23—C22	0.3 (3)
O1—C1—C6—C7	−1.1 (3)	C17—C18—C23—C22	177.69 (19)

### Hydrogen-bond geometry (Å, °)

**Table:**

*D*—H···*A*	*D*—H	H···*A*	*D*···*A*	*D*—H···*A*
O7—H2W···O2^i^	0.77	2.31	3.074 (2)	172
O7—H1W···O4^ii^	0.84	1.89	2.710 (2)	166

Symmetry codes: (i) −*x*−1/2, *y*+1/2, −*z*+1/2; (ii) −*x*+1/2, *y*+1/2, −*z*+1/2.
